# Effects of Thermally-Assisted and High-Pressure Processing on Background Microbiota and the *Listeria monocytogenes* Load of a Minimally Processed Commodity

**DOI:** 10.3390/microorganisms12091858

**Published:** 2024-09-07

**Authors:** Ranju Kafle, Aliyar Cyrus Fouladkhah

**Affiliations:** 1 Public Health Microbiology Laboratory, Tennessee State University, Nashville, TN 37209, USA; rkafle@my.tnstate.edu; 2 Public Health Microbiology FoundationSM, Nashville, TN 37209, USA

**Keywords:** high-pressure processing, *Listeria monocytogenes*, background microbiota

## Abstract

The current study investigated the impact of treatments with elevated hydrostatic pressure (500 MPa) for inactivation of *Listeria monocytogenes* on smoked rainbow trout (*Oncorhynchus mykiss*) at high and low inoculation levels. The temperature values of the trials were set at 4.4 and 60.0 °C, adjusted with a circulating water bath connected to a stainless steel jacket surrounding the pressure processing chamber. Before pressure processing, the counts (selective counts of PALCAM, mean ± SD) of *L. monocytogenes* were 6.45 ± 0.1 log CFU/g and were reduced (*p* < 0.05) to 3.72 ± 0.3, and <1.48 ± 0.8 log CFU/g after 10 min of treatment at 4.4 and 60.0 °C, respectively. Treatments of low inoculation level samples were similarly efficacious and resulted in a reduction (*p* < 0.05) of the pathogen to 1.62 ± 0.3 and <0.82 ± 0.0 log CFU/g for treatments at 4.4 and 60.0 °C, respectively. At 4.4 °C, linear D-value and non-linear k_max1_ were 8.68 and 0.50, and 5.81 and 2.41 for high-inoculation and low-inoculation samples, respectively. Application of hydrostatic pressure at 500 MPa at cold and elevated temperatures was efficacious for up to 5.03 log CFU/g reduction of *L. monocytogenes,* illustrating the potential for further adaptation of this technology.

## 1. Introduction

As a Gram-positive and non-spore-forming bacterium, *Listeria monocytogenes* is an important pathogen of public health concern. The bacterium can survive and multiply in the presence of diverse intrinsic and extrinsic factors of food including pH ranges of 4.0 to 9.5 and temperatures of 0 to 45 °C [1,2,3]. Human illness caused by this pathogen is almost exclusively associated with contaminated food products, with around 99% of listeriosis cases in the United States being categorized as foodborne diseases [1]. Although the pathogen is ubiquitous in nature and can be found in a wide array of food products, it is of major concern for various raw, minimally processed, and ready-to-eat commodities, such as smoked fish [2]. Listeriosis, the disease caused by ingestion of *L. monocytogenes*, is an important public health concern for all age groups, with hospitalization and death rates of 94.0 and 15.9%, respectively [1]. Pregnant women, the very young, the elderly, and the immunocompromised are at elevated risk of developing more severe complications after exposure to the pathogen [3,4]. Typical symptoms of listeriosis can range from mild gastrointestinal discomfort, fever, muscle aches, nausea, and vomiting to more severe outcomes such as septicemia, meningitis, and death [5,6]. This ubiquitous pathogen can be found in an array of locations including domestic environments, food manufacturing facilities, moist environments, decaying vegetation, and soil. The pathogen can contaminate a product without causing any noticeable change in the organoleptic properties of the product and thus consumers may not be able to differentiate between pathogen-free and contaminated products by visual inspection or taste. This pathogen is of particular concern in ready-to-eat foods (RTE) and can be introduced to the product by cross-contamination after processing and before final packaging. Considering the ability of the pathogen to multiply even at refrigerated temperatures, some regulatory agencies in North America have zero tolerance regulations for presence of *L. monocytogenes* in RTE products [2,5]. Regulations in various regions of the world, understandably, vary for testing and presence of this pathogen in various minimally-processed commodities [5].

As a genetically diverse pathogen, *L. monocytogenes* is divided into fourteen serotypes and four genomic lineages of I, II, III, and IV. Human listeriosis cases are mostly associated with lineages I and II, especially serotypes 1/2b, 3b, and most strains of serotype 4b in lineage I, along with serotypes 1/2a, 1/2c, 3a, and 3c in lineage II. These serotypes are associated with severe foodborne illnesses, hospitalizations, and fatalities, further emphasizing the importance of robust food safety measures to protect general and particularly vulnerable populations [7,8].

As discussed earlier, *L. monocytogenes* has the capability of multiplication at refrigeration temperatures and smoked fish products can have a shelf-life of up to 4 weeks and may be consumed without any further thermal processing by consumers [9]. Thus, considering their popularity and minimally-processed nature, smoked fish products can be an important vehicle for this pathogen of public health concern in the food chain [10]. In the European Union, as an example, it is estimated that 1.7% of fish products have more than 100 CFU/g of *L. monocytogenes* at the end of their shelf-lives, surpassing other RTE products such as RTE meats and cheeses. It is noteworthy that, although the infective dose of this pathogen is unknown and could be variable for different individuals, ingestion of as few as 1000 cells of *L. monocytogenes* may cause listeriosis in susceptible individuals [5,11], thus consumption of products with less than 100 CFU/g of pathogen could be potentially hazardous as well, particularly for susceptible individuals. Similar outbreaks have been reported in the United States [5]. Most recently, in 2022–2023, a multi-country outbreak of listeriosis was linked to contaminated smoked fish affecting 17 cases in Austria, Belgium, Italy, Germany, and the Netherlands [12]. Due to their minimally-processed nature, smoked fish products can thus be an important public health vehicle for this ubiquitous and important foodborne pathogen [5,12].

Among various thermal and non-thermal processing technologies for ensuring the safety of food commodities, the application of elevated hydrostatic pressure is gaining popularity in food commerce due to consumer acceptability and commercial feasibility of the technology [13,14]. This technology utilizes elevated hydrostatic pressure at levels typically from 100 to 800 MPa for ensuring the microbial safety of various food commodities including ready-to-eat products [15,16]. To ensure decontamination efficacy and preserve the quality and organoleptic properties of products, the application of multiple hurdle technology is preferred, where instead of reliance on one treatment as a hurdle, a combination of them is utilized to ensure the safety and quality of processed commodities [17,18].

The purpose of the current study was to investigate the impact of elevated hydrostatic pressure on the inactivation of *L. monocytogenes* (low and high inoculation levels) in smoked fish. The efficacy of the treatment was investigated alone and in combination with mild heat as an additional hurdle. Additionally, the impact of pressure-based and thermally-assisted pressure-based treatments was investigated for the reduction of the background microbiota of the product.

## 2. Materials and Methods

### 2.1. Bacterial Cell Preparation

Five strains of *L. monocytogenes*, preserved in −80 °C glycerol stock, were used in this study; they were identified by ATCC^®^ numbers 51772^TM^, 51779^TM^, BAA-2658^TM^, 13932^TM^, and BAA-751^TM^, belonging to serotypes 1/2a, 1/2c, 1/2b, 4b, and 1/2b, respectively. These strains from diverse lineages and ribotypes were selected based on preliminary trials completed in the Public Health Microbiology program in Nashville and based on their epidemiological and public health significance [15,19]. For preparation of the strains for the trials, a loopful of each strain of *L. monocytogenes* was aseptically transferred into tryptic soy broth (TSB) (Difco, Becton Dickinson, Franklin Lakes, NJ, USA) supplemented with 0.6% yeast extract (YE) and subjected to 24-h incubation at 37 °C. Subsequently, each strain from the aforementioned overnight suspension was transferred and spread-plated onto tryptic soy agar containing 0.6% yeast extract (TSAYE) and they were incubated (each strain separately) at 37 °C for 22–24 h. These plates were then stored at refrigerated temperature for up to one month prior to initiation of the trials.

Before the experiments, the inoculum for the trials was prepared by aseptically transferring a single colony from the aforementioned plates and suspending it in tryptic soy broth supplemented with 0.6% yeast extract (TSBYE). After the incubation period, samples were vortexed and a 100 µL aliquot was then aseptically sub-cultured into another 10 mL of TSBYE and incubated again at 37 °C for an additional 22–24 h. After the incubation period, bacterial cells of each strain were harvested via centrifugation at 6000 revolutions per min (3548× *g*, for 88 mm rotor) for 15 min (Model 5424, Eppendorf North America, Hauppauge, NY, USA; Rotor FA-45-24-11) and purified with phosphate buffered saline (PBS, VWR International, Radnor, PA, USA) to eliminate any extraneous components. The resulting purified strains were then combined to make a 5-strain inoculum. The 5-strain cocktail was then serially 10-fold diluted in PBS to achieve a target inoculation level of 6 to 7 log CFU/g (high inoculation) and 4 to 5 log CFU/g (low inoculation) of the final product.

### 2.2. Sample Preparation, Inoculation, and Thermally-Assisted and High-Pressure Processing

For sample preparation, cold-smoked rainbow trout (*Oncorhynchus mykiss*) was obtained from a local supermarket in Nashville, Tennessee. The product was farm-raised fish, harvested in early fall and cold smoked with final sodium content of approximately 1017 mg/100 g of the product. Skinless fillets were cut using sterilized knives into 1.5-g pieces and were aseptically inserted into the no-disk PULSE tubes (Pressure BioScience Inc., South Easton, MA, USA). This volume allows very precise control of temperature and pressure, enabling a concept to be tested with very high internal validity. To reach two target inoculation levels separately, the packed tubes were then inoculated with 100 µL of the above-mentioned 5-strain *L. monocytogenes* inoculum. The inoculation levels were designed for assimilation of the pathogen cross-contamination of the product after production and prior to final packaging. The inoculated tubes were then habituated for 48 h at 4.4 °C to allow for acclimatization of the pathogen cells to the food environment and temperature [20,21]. High-pressure treatments were conducted using the Pressure Bioscience Inc. Hub880 Explorer unit. High-pressure treatments were applied on inoculated and habituated PULSE tubes at 500 MPa, at 4.4 and 60.0 °C for time intervals of 0 min (untreated control), 1, 3, 5, and 10 min. As further detailed in the discussion section, a temperature of 60.0 °C can be considered a mild temperature without major pathogen reduction efficacy alone and hence this temperature was only studied to augment the decontamination efficacy of high-pressure processing. To maintain the desired temperature throughout the experiment, a water jacket made of stainless steel was used to surround the pressure processing chamber that was connected to a refrigerated circulating water bath (Model 160s, VWR International, Radnor, PA, USA). Two K-type thermocouples, inserted into the chamber wall, were then used to monitor the temperature, which was recorded using HUB PBI 2.3.11 Software (Pressure BioScience Inc., South Easton, MA, USA). Thermocouples were firmly secured with thermal paste (Model 5 AS5-3.5G, Arctic Silver, Visalia, CA, USA) for maximizing thermal conductivity between the thermocouples and the chamber wall, ensuring efficient temperature sensing.

### 2.3. Microbial and Physiochemical Analyses

After pressure-based treatments, samples were aseptically removed from the PULSE tubes and transferred into a sterile filtered bag (Whirl-Pak, Nasco, Modesto, CA, USA) with 10 mL of D/E neutralizing broth (Difco, Becton Dickinson, Sparks, MD, USA) and homogenized (200 RPM for 2 min) by a masticator. Samples treated at elevated temperatures were first immediately placed into an ice-water slurry before neutralization and mastication. After homogenization, samples were then 10-fold serially diluted using maximum recovery diluent (Difco, Becton Dickinson, Franklin Lakes, NJ, USA) and thoroughly mixed by vortexing (Scientific Industries Model Vortex-2 Genie, Bohemia, NY, USA). For enumeration of the pathogen, selective medium of Polymyxin Acriflavin Lithium-chloride Ceftazidime Esculin Mannitol (PALCAM) supplemented with Ceftazidime (Becton, Dickinson and Company, Sparks, MD, USA) were used [20]. For the enumeration of background microbiota, tryptic soy agar with 0.6% yeast extract (TSAYE) was utilized as a non-selective medium. TSAYE is a nutrient-rich medium supporting the multiplication of various microorganisms, allowing for the enumeration of total mesophilic aerobic bacteria in the sample [19]. The spread-plated plates were then incubated at 37 °C. After 48 h of incubation, developed colonies were counted manually using the Quebec colony counter. Calculation and log conversions were based on the Bacteriological Analytical Methods (BAM) of the U.S. Food and Drug Administration. To confirm that the inoculated pathogen multiplied as typical *L. monocytogenes* on PALCAM plates were in fact the pathogen and to ensure the product was *L. monocytogenes*-free before inoculation, a multiplex real-time PCR assay (BAX System Q7, Hygiena, Camarillo, CA, USA) was used for confirmation. Water activity (Lab Swift water activity meter, Neutec Group Inc., Farmingdale, NY, USA), pH values (Mettler Toledo AG, Grelfensee, Switzerland), as well as L* (lightness), a* (redness), and b* (yellowness) were measured and remained unchanged (*p* > 0.05) before and after the treatments.

### 2.4. Design, Descriptive and Inferential Statistics

**Figure 1:**
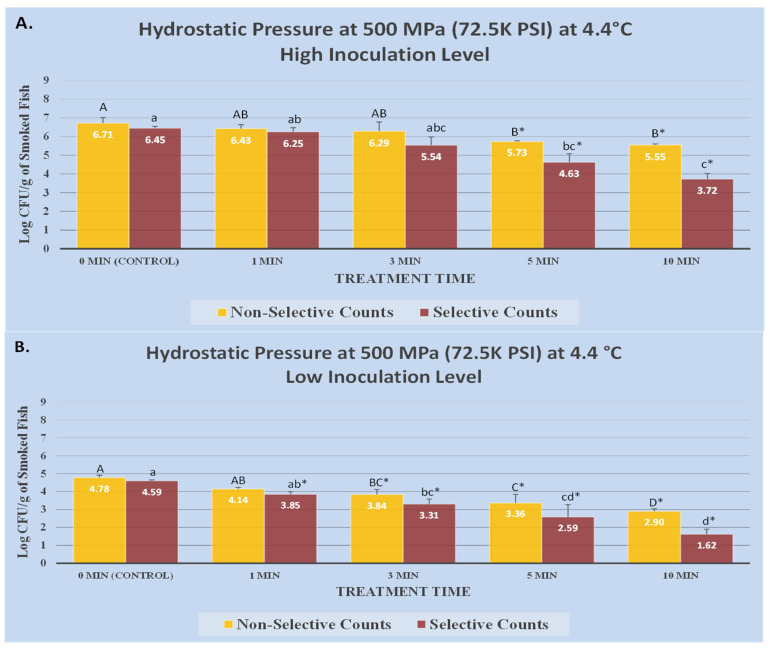
Sensitivity of 5-strain mixture of *L. monocytogenes* (ATCC^®^ numbers 51772 [serotype 1/2a], 51779 [serotype 1/2c], BAA-2658 [serotype 1/2b], 13932 [serotype 4b], BAA-751 [serotype 1/2b]) to elevated hydrostatic pressures, generated by Hub880 Barocycler unit (Bioscience Inc., South Easton, MA, USA). Statistical analyses were conducted for selective (PALCAM) and non-selective (tryptic soy agar supplemented with 0.6% yeast extract) media separately. Non-selective counts marked by different uppercase letters are statistically different (*p* < 0.05) from each other. Selective counts marked by different lowercase letters are statistically different (*p* < 0.05) from each other (Tukey-adjusted paired comparisons at a type I error level of 5%). Additionally, for both media, columns marked by “*” are statistically different (*p* < 0.05) from the untreated control (Dunnett-adjusted mean separation at type I error level of 5%). (**A**) High inoculation samples were treated at 500 MPa at 4.4 °C. (**B**) Low inoculation samples were treated at 500 MPa at 4.4 °C.

**Figure 2:**
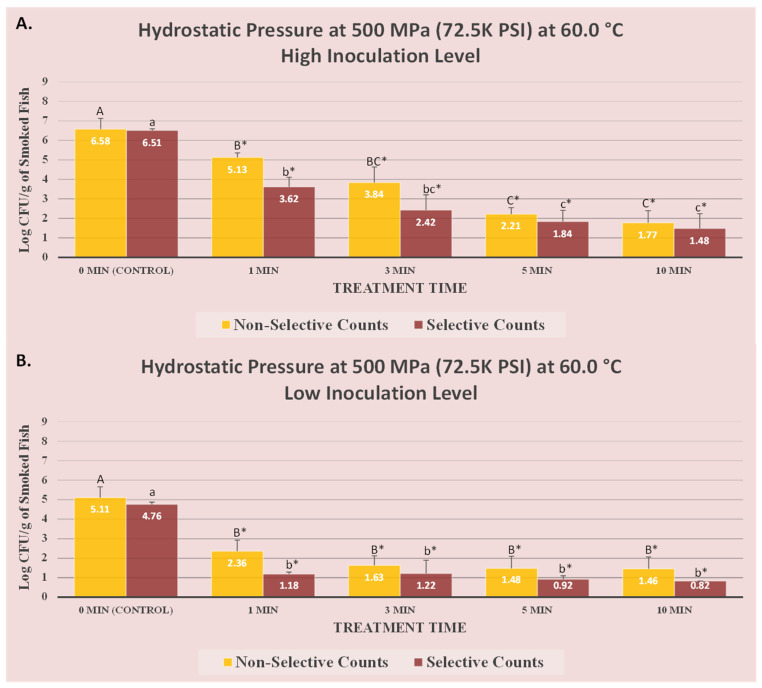
Sensitivity of 5-strain mixture of *L. monocytogenes* (ATCC^®^ numbers 51772 [serotype 1/2a], 51779 [serotype 1/2c], BAA-2658 [serotype 1/2b], 13932 [serotype 4b], BAA-751 [serotype 1/2b]) to elevated hydrostatic pressures, generated by Hub880 Barocycler unit (Bioscience Inc., South Easton, MA, USA). Statistical analyses were conducted for selective (PALCAM) and non-selective (tryptic soy agar supplemented with 0.6% yeast extract) media separately. Non-selective counts marked by different uppercase letters are statistically different (*p* < 0.05) from each other. Selective counts marked by different lowercase letters are statistically different (*p* < 0.05) from each other (Tukey-adjusted paired comparisons at a type I error level of 5%). Additionally, for both media, columns marked by “*” are statistically different (*p* < 0.05) from the untreated control (Dunnett-adjusted mean separation at type I error level of 5%). (**A**) High inoculation samples were treated at 500 MPa at 60.0 °C. (**B**) Low inoculation samples were treated at 500 MPa at 60.0 °C.

This study is a complete randomized block design consisting of two blocks. Each block was a biologically independent trial and each of these blocks consisted of three instrumental replications. Additionally, each of these replications consisted of two microbiological repetitions. Thus, the presented values in Figure 1 and Figure 2 consist of the mean of 12 repetitions (2 blocks × 3 instrumental replications × 2 microbiological repetitions). Counts of background microbiota and *L. monocytogenes* were obtained from TSAYE (representing existing background microbiota) and PALCAM media (representing inoculated pathogen), respectively, and were analyzed and reported separately. Data management, initial descriptive statistics, and log conversion of microbial counts were conducted using Microsoft Excel (2021 Microsoft 365, Microsoft Corporation, Redmond, WA, USA). The tests for normality and homogeneity of variance were conducted on log-normal data before choosing the parametric tests. For inferential statistics, the log-transformed microbial count underwent statistical analyses using a generalized linear model in SAS (version 9.4, SAS Institute, Cary, NC, USA). Tukey-adjusted ANOVA was employed for the mean separation of samples, enabling pair-wise comparisons. Additionally, Dunnett-adjusted ANOVA was utilized for specific comparisons between treatments and controls. The significance level for these comparisons was 5% (alpha = 0.05). Inactivation indices, D-value, and K_max_ were computed using Microsoft Excel and GInaFiT (version 1.6, Katholieke Universiteit, Leuven, Belgium).

## 3. Results and Discussion

The current study investigated the impact of high-pressure processing at 4.4 and 60.0 °C. These temperatures are considered as boundaries of the USDA FSIS Danger Zone, as a recommendation to the food industry to avoid time-temperature abuse of food products [22]. However, it is noteworthy that the temperature of 60 °C is not necessarily sufficient for thermal processing to ensure the safety of a product. This study used this temperature as a hurdle in the context of multiple hurdle technology for ensuring the safety of the product [17,23]. This temperature is considered lower than typical pasteurization temperatures; as an example, fluid milk is heated to at least 72 °C for 15 s during pasteurization [24], and according to the U.S. Food and Drug Administration, food containing raw shell eggs (broken for immediate use) will need to be heated to 68 °C for 17 s [25]. Others have similarly shown that a product pasteurized at 62.8 °C (minimum long-time low-temperature pasteurization temperature) cannot inhibit *L. monocytogenes* and this pathogen can potentially survive such treatment [26].

The current study investigated the inactivation of this important pathogen of public health concern both at high inoculation (target 6 to 7 log CFU/g) and low inoculation (target inoculation of 4 to 5 log CFU/g) levels to provide more comprehensive results discussing the efficacy of pressure-based treatments against *L. monocytogenes*. Additionally, the study was conducted to evaluate the impact of the treatment on the background microbiota of the product in addition to the inoculated pathogen. The detection limit of both low- and high-inoculation trials was 0.82 log CFU/g, thus counts below the detection limit were reported as <0.82 log CFU/g. It is noteworthy that this project used inoculated *L. monocytogenes* and naturally occurring background microbiota of the product. To increase the external validity of the trials and to ensure that the inoculated pathogen had acclimated to the food environment, the inoculated product was habituated for 48 h prior to trial, as further detailed in Section 2.2.

### 3.1. Impact of High-Pressure Processing for Inactivation of L. monocytogenes

Treatments at the temperature of 4.4 °C and intensity level of 500 MPa were effective (*p* < 0.05) for inactivation of inoculated *L. monocytogenes* and background microbiota (Figure 1A). Before the treatment and at high inoculation level, *L. monocytogenes* counts were 6.45 ± 0.1 log CFU/g. After 1, 3, 5, and 10 min of treatment, the pathogen counts were 6.25 ± 0.2, 5.54 ± 0.4, 4.63 ± 0.5, and 3.72 ± 0.3 log CFU/g, respectively (Figure 1A). As a result of these treatments, 5- and 10-min samples were reduced (*p* < 0.05) by 1.8, and 2.7 log CFU/g, respectively (Figure 1A). For high inoculation level samples, counts of background microbiota of the product were similarly affected at this temperature and pressure (Figure 1A). The count before treatment was 6.71 ± 0.3 log CFU/g. While treatment at 4.4 °C/500 MPa was not effective (*p* ≥ 0.5) for 1 min, the treatment was efficacious (*p* < 0.05) in reducing the background microbiota after 3, 5, and 10 min. Counts of background microbiota were reduced (*p* < 0.05) by 0.4, 1.0, and 1.2 log CFU/g after 3, 5, and 10 min of treatment, respectively (Figure 1A). It is noteworthy that reduction of background microbiota was more modest, relative to reductions associated with the pathogen. As an example, log reductions associated with *L. monocytogenes* and background microbiota after 10 min of treatment at 4.4 °C/500 MPa were 2.7 and 1.2 log CFU/g, respectively (Figure 1A). This finding is in harmony with published literature, as background microbiota typically consist of some spore-forming microorganisms, and endospores are inherently more resistant to elevated hydrostatic pressure [15,27].

Treatments at low inoculation levels were similarly effective (*p* < 0.05) for reduction of the pathogen and background microbiota counts of the product. At low inoculation level, these counts were 4.59 ± 0.0 and 4.78 ± 0.1 log CFU/g, respectively (Figure 1B) before treatment. Log reductions associated with the pathogen treated at 4.4 °C/500 MPa for 1, 3, 5, and 10 min were 0.7, 1.3, 2.0, and 3.0 log CFU/g, respectively, while the corresponding reductions for background microbiota were 0.6, 0.9, 1.4, and 1.9 log CFU/g, respectively (Figure 1B). Trends associated with background microbiota and pathogen reductions were very similar, since the high-pressure processing method is a physical decontamination method. However, similar to the results discussed above from Figure 1A, background microbiota exhibited higher resistance to treatments relative to *L. monocytogenes* (Figure 1B). As discussed earlier, this could be attributed to the presence of spore-forming microorganisms and bacterial endospores as part of the background microbiota [15,27].

### 3.2. Impact of Thermally-Assisted High-Pressure Processing for Inactivation of L. monocytogenes

Treatments at the mild temperature of 60.0 °C and 500 MPa were considerably more effective for the inactivation of the pathogen and background microbiota compared with treatments at 4.4 °C/500 MPa (Figure 2A). At high inoculation level, background microbiota and *L. monocytogenes* counts were 6.58 ± 0.5 and 6.51 ± 0.1 log CFU/g (Figure 2A). For both counts, even 1 min of treatment at 60.0 °C/500 MPa was effective (*p* < 0.05) for reducing the background microbiota and the pathogen counts by 1.5 and 2.9 log CFU/g, respectively (Figure 2A). The corresponding log reduction for treatments at 4.4 °C/500 MPa were only 0.3 and 0.2 log CFU/g, respectively (Figure 1A). This highlights the major improvement in efficiency of pressure-based inactivation of the pathogen and spoilage organisms in the presence of mild heat as an additional hurdle for decontamination. Treatments at 3, 5, and 10 min at 60.0 °C/500 MPa were able to reduce (*p* < 0.05) 2.7, 4.4, and 4.8 log CFU/g of background microbiota and 2.9, 4.1, and 4.7 log CFU/g of inoculated *L. monocytogenes*, respectively (Figure 2A). The impact of mild heat for augmenting the decontamination efficacy of pressure-based pasteurization was even more pronounced for the inactivation of background microbiota, the main cause of spoilage in the products. As an example, 10 min of treatment at 4.4 °C/500 MPa was able to eliminate slightly more than 90% (i.e., 1.2 log CFU/g) of the background microbiota, while the same treatment at 60.0 °C resulted in nearly 99.999% (i.e., 4.8 log CFU/g) reduction (Figure 2A). This notable impact of mild heat to augment the efficacy of high-pressure processing has been previously reported in the literature for trials conducted under similar conditions [19].

At low inoculation level, similar results were observed at 60.0 °C/500 MPa, with 1, 3, 5, and 10 min resulting in 3.6, 3.5, 3.8, and 3.9 log CFU/g reduction (*p* < 0.05) of inoculated *L. monocytogenes* (Figure 2B), respectively. A reduction of 2.8, 3.5, 3.6, and 3.7 was similarly observed after the treatments of background microbiota for 1, 3, 5, and 10 min at 60.0 °C/500 MPa, respectively. These results further confirm the efficacy of high-pressure and thermally-assisted high-pressure treatments for reducing this microbial pathogen of public health concern and extending the shelf life of the product. The impact of elevated hydrostatic pressure was exhibited in the past for extending the shelf-life of products similar to the product used in this study, including oysters [28], shrimps, clams [29], and salmon roe [30]. The vast majority of current pressure-treated products in the market are processed at 600 MPa and thus the use of 500 MPa treatment, which could be considered as a mild pressure pasteurization, could protect the quality characteristics of the products sensitive to higher levels of elevated hydrostatic pressure [14,15,16].

This could be of particular interest for minimally processed commodities, i.e., those that are only slightly altered so that they can be more easily eaten, stored, or manufactured while their nutritional content is not substantially altered [31]. As discussed earlier, the detection limit of the current study is 0.82 log CFU/g, thus, samples in Figure 2B with counts below this value should be considered as having a microbial load of <0.82 log CFU/g. This approach will ensure that pathogens that are viable but nonculturable (VBNC) are accounted for [32].

Previous studies illustrated comparable results to our findings. As an example, treatment of around 414 MPa for 5 min was reported to cause 4-log reduction of *Listeria innocua* [33]. Similarly, treatment of 600 MPa for up to 5 min was reported to cause >6 log reduction of *L. monocytogenes* in rainbow trout and fresh European catfish fillets [34]. The same level of pressure treatment was reported to protect a fish product against *L. monocytogenes* during the 28 days of storage for unopened containers [35]. Others have reported on use of very mild pressure of 200 MPa for 15 min, as an additional hurdle to existing processing conditions of smoked fish and observed that even this mild level of pressure could lead to significant reduction of *L. monocytogenes* when coupled with other hurdles such as mild heat and smoking [36]. Recent review studies of the impact of high-pressure processing on various commodities additionally discussed the impact of processing conditions for ensuring safety of various RTE and seafood products [37,38]. Future studies in this area could investigate the impact of bioactive compounds such as nisin and their impact on the pressure-stressed microbial survivors of the treatment during the products’ shelf-lives. Application of microscopy on biotic and abiotic surfaces could be a microbial technique of importance for better assimilation of impact of high-pressure processing on planktonic and sessile cells.

### 3.3. Inactivation Indices for Pressure-Based Reduction of L. monocytogenes

While counts of background microbiota (counts obtained from tryptic soy agar supplemented with 0.6% yeast extract) could be of importance from a business perspective i.e., for extension of the product’s shelf life, the pathogen counts are important for meeting the regulatory requirements of intrastate, interstate, and global commerce and from a public health perspective. Thus, inactivation indices are only calculated for the pathogen counts obtained from PALCAM medium [15].

**Figure 3:**
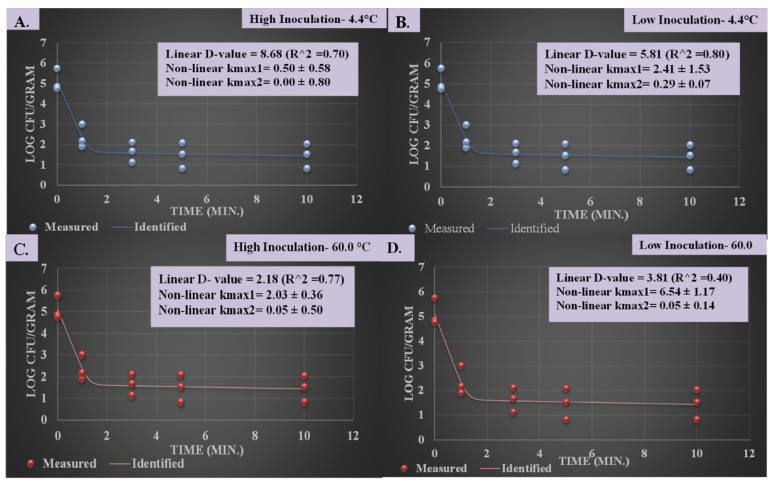
Linear and non-linear inactivation indices associated with high-pressure processing of a 5-strain mixture of *L. monocytogenes* (ATCC^®^ numbers 51772 [serotype 1/2a], 51779 [serotype 1/2c], BAA-2658 [serotype 1/2b], 13932 [serotype 4b], BAA-751 [serotype 1/2b]) inoculated on smoked rainbow trout. D-values were calculated as reciprocal of slope (positive value) of the best-fitted linear model (goodness-of-fit indicator for regression model was R^2^ [α = 0.05]), resulting from plotting log-converted *L. monocytogenes* counts and treatment time (min), thus D-value has the unit of min. The K_max_ values have the unit of 1/min and are derived from non-linear models obtained from GInaFiT software v1.6. K_max_ values are expressions of the numbers of log cycles of *L. monocytogenes* derived from a biphasic curve. (**A**) Samples with high inoculation level treated at 500 MPa at 4.4 °C. (**B**) Samples with low inoculation level treated at 500 MPa at 4.4 °C. (**C**) Samples with high inoculation level treated at 500 MPa at 60.0 °C. (**D**) Samples with low inoculation level treated at 500 MPa at 60.0 °C.

The current study utilized linear and non-linear inactivation indices to illustrate the pathogen reduction capability of the tested conditions (Figure 3A–D). The D-value was calculated based on a linear regression model and is reported with the unit of min. This value shows the number of log reductions that can be achieved per unit of time for the specific product and testing conditions. For high inoculation levels of *L. monocytogenes*, the D-value was 8.68 and 2.18 min for products treated at 500 MPa at 4.4 and 60.0 °C, respectively (Figure 3A,C). This illustrates that every 2.18 min of treatment, with linearity assumption, a treatment of 60.0 °C/500 MPa can eliminate 90% of *L. monocytogenes* in this product while the same treatment of 500 MPa at 4.4 °C requires more than 8 min to achieve the same pathogen reduction outcome. This is in harmony with a previously published study where researchers reported D-values of 9.3 and 1.3 min for orange juice samples treated at 500 MPa for 60.0 and 4.4 °C, respectively [14].

In the current study, as with the results illustrated earlier, at low inoculation levels, D-values of 5.81 and 3.81 min were observed for samples treated at 500 MPa for 4.4 and 60.0 °C, respectively (Figure 3B,D). Similar trends were observed using a non-linear biphasic inactivation model where K_max1_ (unit 1/min) corresponded to the reduction phase of the pathogen/product/treatment. The K_max1_ values were 2.41 ± 1.52 and 0.5 ± 0.58 1/min for low- and high-inoculation samples treated at 4.4 °C/500 MPa, respectively (Figure 3A,B). The corresponding K_max1_ values for samples treated at 60.0 °C/500 MPa were 6.54 ± 1.17 and 0.05 ± 0.50 1/min, respectively (Figure 3C,D).

## 4. Conclusions

Under the condition of the trials, the current study illustrated that high-pressure processing at 4.4 °C/500 MPa could eliminate (*p* < 0.05) up to 99.9% (i.e., 3 log CFU/g) of inoculated *L. monocytogenes* and close to 99% (i.e., 1.9 log CFU/g) of background microbiota of the cold-smoked rainbow trout. The same treatment synergized with mild temperature (i.e., 60.0 °C/500 MPa treatment) was able to reduce (*p* < 0.05) >99.999% of inoculated *L. monocytogenes* (i.e., 5.0 log CFU/g) and close to 99.999% (i.e., 4.8 log CFU/g) of background microbiota. These results illustrate that high-pressure processing and thermally-assisted high-pressure processing can be used effectively for the reduction of pathogens of public health concern and reducing background microbiota for extension of this product’s shelf life. The application of mild heat (60.0 °C) as an additional hurdle in the context of multiple hurdle technology was able to a great extent augment the pathogen reduction efficacy of the elevated hydrostatic pressure. The combination of mild heat and pressure could assist manufacturers of products similar to this product in mitigating quality and organoleptic issues associated with the use of extreme heat and/or pressure treatments and assist in extending the products’ shelf lives, thus illustrating the potential for further adaptation of this technology in ready-to-eat and minimally processed products.

## Data Availability

The datasets of the current study can be obtained by contacting the study’s corresponding author with reasonable requests. A request can be submitted by obtaining the contact information from the Public Health Microbiology Foundation^SM^ at https://publichealthmicrobiology.education/ (accessed on 6 September 2024). The SAS codes used for statistical analyses in the current study were derived from no-cost and publicly available sources with needed modifications and can be obtained by contacting the study’s corresponding author with reasonable requests.
